# Influenza Vaccination Coverage Among Health Care Personnel — United States, 2016–17 Influenza Season

**DOI:** 10.15585/mmwr.mm6638a1

**Published:** 2017-09-29

**Authors:** Carla L. Black, Xin Yue, MPS, Sarah W. Ball, Rebecca Fink, Marie A. de Perio, A. Scott Laney, Walter W. Williams, Megan C. Lindley, Samuel B. Graitcer, Peng-Jun Lu, Rebecca Devlin, Stacie M. Greby

**Affiliations:** ^1^Immunization Services Division, National Center for Immunization and Respiratory Diseases, CDC; ^2^Abt Associates Inc., Cambridge, Massachusetts; ^3^Division of Surveillance, Hazard Evaluations, and Field Studies, National Institute for Occupational Safety and Health, CDC; ^4^Division of Respiratory Health, National Institute for Occupational Safety and Health, CDC.

The Advisory Committee on Immunization Practices (ACIP) recommends that all health care personnel (HCP) receive an annual influenza vaccination to reduce influenza-related morbidity and mortality among HCP and their patients and to reduce absenteeism among HCP (*1*–*4*). To estimate influenza vaccination coverage among HCP in the United States during the 2016–17 influenza season, CDC conducted an opt-in Internet panel survey of 2,438 HCP. Overall, 78.6% of survey respondents reported receiving vaccination during the 2016–17 season, similar to reported coverage in the previous three influenza seasons (*5*). Vaccination coverage continued to be higher among HCP working in hospitals (92.3%) and lower among HCP working in ambulatory (76.1%) and long-term care (LTC) (68.0%) settings. As in previous seasons, coverage was highest among HCP who were required by their employer to be vaccinated (96.7%) and lowest among HCP working in settings where vaccination was not required, promoted, or offered on-site (45.8%). Implementing workplace strategies found to improve vaccination coverage among HCP, including vaccination requirements or active promotion of on-site vaccinations at no cost, can help ensure that HCP and patients are protected against influenza (*6*).

The Internet panel survey of HCP was conducted for CDC by Abt Associates, Inc. (Cambridge, Massachusetts) during March 28–April 19, 2017, to provide estimates of influenza vaccination coverage during the 2016–17 influenza season. Similar surveys have been conducted since the 2010–11 influenza season, and survey methodology has been described previously (*7*). HCP were recruited from two preexisting national opt-in Internet sources: Medscape, a medical website managed by WebMD Health Professional Network,* and general population Internet panels operated by Survey Sampling International (SSI).^†^ Responses were weighted to the distribution of the U.S. population of health care personnel by occupation, age, sex, race/ethnicity, work setting, and U.S. Census region.^§^ Because the study sample was based on HCP from opt-in Internet panels rather than probability samples, statistical testing was not conducted.^¶^ An increase or decrease of at least 5 percentage points between seasonal estimates was considered a change; estimates with smaller differences were considered similar.

Among the 2,547 HCP who started the survey from either Medscape or SSI and had eligible responses to the screening questions, 2,493 (97.9%) completed the survey.** Fifty-four respondents with completed surveys who reported working in “other health care settings” were excluded because examination of their survey responses indicated that they were either unlikely to have contact with patients or to have worked in one of the health care settings of interest for this analysis; in addition, one respondent whose work location was in Canada was excluded. The final analytic sample consisted of 2,438 HCP.

Overall, 78.6% of respondents reported having received an influenza vaccination during the 2016–17 season. Among all HCP, coverage increased from 63.5% in the 2010–11 season to 75.2% in the 2013–14 season, and ranged from 77.3% to 79.0% in subsequent seasons (Figure) (Table 1). As in previous surveys, coverage in the 2016–17 season was highest among HCP working in hospital settings (92.3%), followed by HCP working in ambulatory care (76.1%), other clinical settings (75.0%), or LTC (68.0%) settings. Coverage among HCP working in other clinical settings increased from 69.8% in 2015–16 to 75.0% in 2016–17; coverage in hospital, ambulatory care, and LTC settings was similar in 2015–16 and 2016–17 (Table 1). Among vaccinated HCP, 76.5% were vaccinated at their workplace.

**FIGURE:**
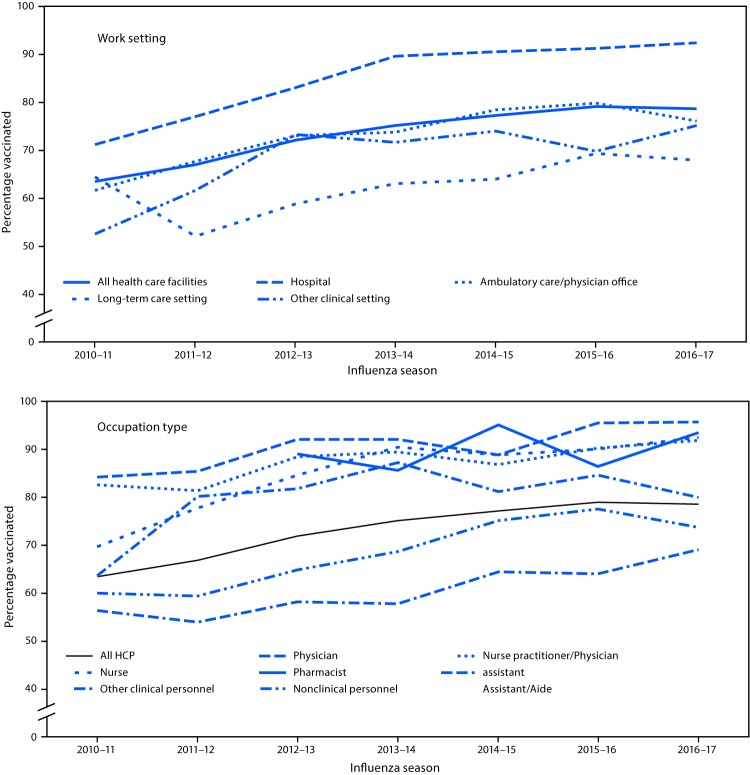
Percentage of health care personnel (HCP) who reported receiving influenza vaccination, by work setting* and occupation type^†^— Internet panel surveys, United States, 2010–11 through 2016–17 influenza seasons * Respondents could select more than one work setting. The “ambulatory care/physician office” category includes physician’s office, medical clinic, and other ambulatory care setting. The “other clinical setting” category includes dentist office or dental clinic, pharmacy, laboratory, public health setting, emergency medical services setting, or other setting where clinical care or related services were provided to patients. ^†^ For the 2010–11 influenza season, dentists were included in the physician category. Before the 2012–13 season, separate data on pharmacists were not collected. The “other clinical personnel” category includes allied health professionals, technicians, and technologists. The “nonclinical personnel” category includes administrative support staff members or managers and nonclinical support staff members (e.g., food service workers, laundry workers, janitors, and other members of the housekeeping and maintenance staffs).

**TABLE 1:** Percentage of health care personnel* (HCP) who reported receiving influenza vaccination, by work setting and occupation type — Internet panel surveys, United States, 2015–16 and 2016–17 influenza seasons

Work setting/Occupation type^†^	2015–16 season			2016–17 season			Percentage-point difference (2015–16 to 2016–17)
	No. in sample	Weighted %^§^	Weighted % vaccinated	No. in sample	Weighted %^§^	Weighted % vaccinated	
**Overall**	**2,258**	**100**	**79.0**	**2,438**	**100**	**78.6**	**-0.4**
**Occupational setting, by occupation**							
**Hospital**	**803**	**39.7**	**91.2**	**925**	**40.5**	**92.3**	**1.1**
Physician	127	3.7	99.4	129	4.2	97.8	−1.6
NP/PA	50	0.9	90.0	57	0.8	94.6	4.6
Nurse	95	23.8	94.6	108	28.5	96.4	1.8
Pharmacist	16	0.7	—^¶^	121	1.2	97.4	—
Assistant/Aide	107	8.9	88.2	118	8.4	91.1	2.9
Other clinical HCP**	236	23.4	94.4	232	22.0	90.0	−4.4
Nonclinical HCP^††^	155	38.2	87.2	144	34.6	89.7	2.5
**Ambulatory care/Physician office** ^§§^	**648**	**27.6**	**79.8**	**718**	**28.8**	**76.1**	**-3.7**
Physician	216	10.4	95.2	198	9.5	94.8	−0.4
NP/PA	92	2.4	89.1	110	2.7	90.0	0.9
Nurse	45	20.6	88.6	48	20.5	93.3	4.7
Pharmacist	6	0.4	—^¶^	24	0.3	—^¶^	—
Assistant/Aide	57	9.2	62.0	74	9.5	74.4	12.4
Other clinical HCP**	135	22.0	81.7	139	22.9	71.1	−10.6
Nonclinical HCP^††^	91	34.8	72.9	111	34.1	63.0	−9.9
**Long-term care setting**	**659**	**29.6**	**69.2**	**549**	**29.3**	**68.0**	**-1.2**
Physician	17	0.8	—^¶^	15	0.7	—^¶^	—
NP/PA	7	0.2	—^¶^	7	0.2	—^¶^	—
Nurse	23	9.6	—^¶^	22	9.7	—^¶^	—
Pharmacist	1	0	—^¶^	6	0.1	—^¶^	—
Assistant/Aide	501	58.4	61.9	428	59.3	66.9	5.0
Other clinical HCP**	54	7.6	85.9	26	3.8	—^¶^	—
Nonclinical HCP^††^	54	23.3	70.9	44	26.3	60.7	−10.2
**Other clinical setting** ^¶¶^	**409**	**11.6**	**69.8**	**604**	**12.6**	**75.0**	**5.2**
Physician	4	0.6	—^¶^	4	0.4	—^¶^	—
NP/PA	5	0.3	—^¶^	6	0.3	—^¶^	—
Nurse	15	15.2	—^¶^	15	15.3	—^¶^	—
Pharmacist	51	9.5	85.5	243	8.7	92.4	6.9
Assistant/Aide	42	15.4	51.2	54	15.2	63.1	11.9
Other clinical HCP**	257	32.9	72.5	240	35.3	76.5	4.0
Nonclinical HCP^††^	22	25.3	—^¶^	31	24.0	69.6	—
**Overall occupation**							
Physician	284	3.6	95.6	251	3.4	95.8	0.2
NP/PA	134	1.0	90.3	154	1.0	92.0	1.7
Nurse	168	18.5	90.1	167	18.6	92.6	2.5
Pharmacist	63	1.3	86.5	307	1.3	93.7	7.2
Assistant/Aide	673	23.8	64.1	641	23.9	69.1	5.0
Other clinical HCP**	599	18.8	84.7	572	18.9	80.0	−4.7
Nonclinical HCP^††^	307	32.9	77.7	315	32.6	73.7	−4.0

**Abbreviations:** NP = nurse practitioner; PA = physician assistant.

* Persons who worked in a place where clinical care or related services were provided to patients, or whose work involved face-to-face contact with patients or who were ever in the same room as patients.

^†^ Respondents could specify working in more than one setting.

^§^ Weights were calculated based on each occupation type, by age, sex, race/ethnicity, work setting, and U.S. Census region to represent the U.S. population of HCP. Work setting and overall occupation are presented as weighted estimates of the total sample. Where the groups are stratified by work setting, the estimates are presented as weighted estimates of the occupation group subsample of each work setting subgroup.

^¶^ Vaccination coverage estimate not reliable because the sample size was <30.

** Allied health professional, technician, or technologist.

^††^ Administrative support staff members or manager and nonclinical support staff members (including food service workers, laundry workers, janitors, and members of the housekeeping and maintenance staffs).

^§§^ Physician’s office, medical clinic, or other ambulatory care setting.

^¶¶^ Dentist office or dental clinic, pharmacy, laboratory, public health setting, emergency medical services setting, or other setting where clinical care or related services was provided to patients.

Overall, vaccination coverage in 2016–17 was highest among physicians (95.8%), nurse practitioners and physician assistants (92.0%), nurses (92.6%), and pharmacists (93.7%), and lowest among other clinical HCP (80.0%), assistants and aides (69.1%), and nonclinical HCP (73.7%) (Table 1). However, in hospital settings, vaccination coverage was approximately 90% or higher in all occupational groups, including assistants and aides and nonclinical personnel.

Overall, 42.3% of HCP reported a requirement to be vaccinated for the 2016–17 season, an increase over the 2013–14 season but similar to the 2014–15 and 2015–16 seasons. HCP working in hospitals were more likely to report a vaccination requirement (69.5%) than were HCP working in ambulatory care (39.0%), LTC (26.2%), or other clinical settings (22.0%) (Table 2). HCP working in ambulatory care, LTC, and other clinical settings more often reported that their employer did not require, provide, or promote vaccination (21.7%, 30.5%, and 32.2%, respectively), compared with HCP working in hospital settings (3.9%).

**TABLE 2:** Percentage of health care personnel* (HCP) who reported receiving influenza vaccination, by work setting, workplace vaccine availability, and employer vaccine requirements status — Internet panel surveys, United States, 2013–14 through 2016–17 influenza seasons

Characteristic	2013–14 season			2014–15 season			2015–16 season			2016–17 season		
	No. in sample	Weighted %^†^	Weighted % vaccinated	No. in sample	Weighted %^†^	Weighted % vaccinated	No. in sample	Weighted %^†^	Weighted % vaccinated	No. in sample	Weighted %^†^	Weighted % vaccinated
**Employer vaccination requirement** **^§^**	**738**	**35.5**	**97.8**	**725**	**40.1**	**96.0**	**841**	**37.8**	**96.5**	**983**	**42.3**	**96.7**
Hospital	520	58.2	97.7	440	64.8	97.2	510	61.0	96.5	644	69.5	98.3
Ambulatory care/Physician office^¶^	252	33.6	96.4	277	34.7	96.1	258	33.9	98.7	305	39.0	97.2
Long-term care	88	20.1	98.4	104	26.0	97.3	143	23.4	93.8	142	26.2	90.0
Other clinical setting**	88	29.3	99.5	109	35.9	85.7	101	24.9	98.5	135	22.0	98.2
**On-site vaccination >1 day** **^††^**	**542**	**25.1**	**80.4**	**407**	**19.1**	**83.9**	**460**	**19.8**	**82.8**	**434**	**15.2**	**80.3**
Hospital	261	31.4	82.0	151	21.0	86.9	173	23.8	81.8	152	13.8	80.9
Ambulatory care/Physician office^¶^	183	28.6	80.7	165	23.1	87.8	152	20.8	85.1	118	16.6	82.3
Long-term care	63	11.7	71.6	57	12.4	67.3	96	16.1	80.4	61	14.0	76.1
Other clinical setting**	107	22.0	85.0	97	15.6	81.9	87	12.3	84.1	155	15.6	82.8
**On-site vaccination 1 day** **^§§^**	**169**	**7.6**	**61.6**	**230**	**9.8**	**73.6**	**254**	**10.9**	**82.1**	**361**	**14.2**	**73.8**
Hospital	43	4.2	55.6	51	7.3	72.1	70	8.3	81.1	82	10.4	78.3
Ambulatory care/Physician office^¶^	76	11.3	69.3	104	10.9	80.6	76	12.8	82.9	126	16.7	73.2
Long-term care	43	10.0	54.1	45	10.0	67.1	77	11.5	83.0	77	15.6	66.7
Other clinical setting**	31	6.5	72.9	50	10.8	80.4	54	14.2	85.2	111	15.2	78.6
Other vaccination promotion***	**226**	**15.5**	**61.9**	**216**	**12.4**	**59.5**	**293**	**13.0**	**67.8**	**206**	**8.2**	**70.4**
Hospital	46	5.1	80.7	24	4.4	—^¶¶^	39	4.6	91.0	31	2.5	81.8
Ambulatory care/Physician office^¶^	66	12.2	53.5	67	10.3	60.5	62	11.9	74.0	46	6.1	59.6
Long-term care	90	29.8	62.2	83	21.6	58.5	139	21.4	63.4	69	13.7	71.7
Other clinical setting**	50	16.9	57.5	54	14.6	64.5	67	16.4	54.0	77	15.1	76.7
**No requirement, on-site vaccination or promotion**	**207**	**16.3**	**36.8**	**336**	**18.7**	**44.0**	**409**	**18.4**	**44.9**	**454**	**20.0**	**45.8**
Hospital	10	1.2	—^¶¶^	15	2.6	—^¶¶^	11	2.3	—^¶¶^	16	3.9	—^¶¶^
Ambulatory care/Physician office^¶^	72	14.3	26.8	133	21.0	46.6	100	20.6	45.0	123	21.7	40.1
Long-term care	80	28.5	38.6	117	30.0	36.4	204	27.7	40.6	200	30.5	44.3
Other clinical setting**	51	25.3	36.9	79	23.2	53.4	100	32.1	43.4	126	32.2	52.8

* Persons who worked in a place where clinical care or related services were provided to patients, or whose work involved face-to-face contact with patients or who were ever in the same room as patients.

^†^ Weights were calculated based on each occupation type, by age, sex, race/ethnicity, work setting, and U.S. Census region to represent the U.S. population of HCP. Work setting and overall occupation are presented as weighted estimates of the total sample. Where the groups are stratified by work setting, the estimates are presented as weighted estimates of the occupation group subsample of each work setting subgroup.

^§^ Includes all respondents who indicated that their employer required them to be vaccinated for influenza.

^¶^ Physician’s office, medical clinic, or other ambulatory care setting.

** Dentist office or dental clinic, pharmacy, laboratory, public health setting, health care education setting, emergency medical services setting, or other setting where clinical care or related services was provided to patients.

^††^ Employer made influenza vaccination available on-site for >1 day during the influenza season at no cost to employees. Restricted to respondents without an employer requirement for vaccination.

**^§§^** Employer made influenza vaccination available on-site for 1 day during the influenza season at no cost to employees. Restricted to respondents without an employer requirement for vaccination.

^¶¶^ Vaccination coverage estimate not reliable because the sample size was <30.

*** Influenza vaccination was promoted among employees through public identification of vaccinated persons, financial incentives, or rewards to individuals or groups of employees, competition between units or care areas, free or subsidized cost of vaccination, personal reminders to be vaccinated, or publicizing of the number or percentage of employees receiving vaccination. Restricted to respondents without an employer requirement for vaccination or on-site vaccination.

As in previous seasons, vaccination coverage in 2016–17 was highest (96.7%) among HCP working in settings where vaccination was required, ranging from 90.0% in LTC settings to 98.3% in hospital settings (Table 2). Among HCP whose employers did not have a requirement for vaccination, coverage was higher among those who worked in locations where vaccination was available at the worksite at no cost for >1 day (80.3%) than among those with vaccination available for 1 day only (73.8%) or among those who worked in locations where their employer did not provide influenza vaccination on-site at no cost but actively promoted vaccination through other mechanisms^††^ (70.4%). Vaccination coverage was lowest (45.8%) among HCP working in locations where employers did not require vaccination, provide vaccination on-site at no cost, or promote vaccination (Table 2).

## Discussion

The overall influenza vaccination coverage estimate among HCP was 78.6% in the 2016–17 season, an increase of 15 percentage points since the 2010–11 season, but similar to the 2013–14 through 2015–16 seasons (*5*). As in previous seasons, the highest coverage was among HCP whose workplace had vaccination requirements. In the absence of requirements, HCP with vaccination available at their workplace had higher coverage than those without on-site vaccination. HCP working in hospital settings consistently reported higher vaccination coverage than did those working in other settings and were the most likely to report workplace vaccination requirements and on-site vaccination. Even in occupational groups with lower overall coverage (i.e., assistants, aides, and nonclinical personnel), hospital personnel reported vaccination coverage ≥90%. In the 2016–17 season, 93.7% of HCP working in hospital settings reported either having a vaccination requirement or having on-site vaccination for at least 1 day. Most vaccinated HCP reported being vaccinated at their place of work, underscoring the importance of workplace vaccination availability.

HCP working in LTC settings consistently have lower influenza vaccination coverage than do HCP working in all other health care settings. Influenza vaccination among HCP in LTC settings is especially important because influenza vaccine effectiveness is generally lowest in the elderly, who are at increased risk for severe disease (*2*). In addition, studies have demonstrated that vaccination of HCP in LTC settings confers a health benefit to patients, including reduced risk for mortality (*1*–*3*). In contrast to HCP working in hospitals, only 26.2% of respondents working in LTC settings reported having a workplace requirement for vaccination. Among HCP in LTC settings, 30.5% reported that their employer did not require vaccination, make vaccination available on-site at no cost, or promote vaccination in any way. Workplace vaccination programs that have been successful in increasing coverage in hospital settings could be implemented in LTC and other settings with lower vaccination coverage. Outside of hospital settings, assistants and aides, “other” clinical personnel, and nonclinical HCP have persistently low vaccination coverage. Although some facilities might not prioritize these groups for vaccination programs, especially nonclinical HCP, these personnel often spend considerable time with and in proximity to patients.

The findings in this report are subject to at least three limitations. First, the study used a nonprobability sample of volunteer members of Medscape and SSI Internet panels, which might affect the generalizability of these findings to the U.S. population of HCP. Second, vaccination status and vaccination requirements were self-reported and might be subject to recall bias. Finally, coverage findings from Internet survey panels have differed from population-based estimates from the National Health Interview Survey in past influenza seasons, although trends in coverage were similar across seasons (*8*,*9*).

The highest influenza vaccination coverage among HCP continues to be reported in worksites with employer requirements for vaccination. In the absence of vaccination requirements, the findings in this study support the recommendations found in the Guide to Community Preventive Services, which include active promotion of on-site vaccination at no cost or low cost to increase influenza vaccination coverage among HCP (*6*). Measurement of and feedback about vaccination coverage are additional interventions recommended by the Community Preventive Services Task Force (*6*). Federal reporting requirements might influence vaccination coverage by occupational setting (*10*). CDC’s National Healthcare Safety Network (NHSN) has included reporting of health care personnel influenza vaccination since 2012. During 2013–2015, the Centers for Medicare & Medicaid Services (CMS) added requirements to report health care personnel influenza vaccination data through NHSN for acute care hospitals (2013), ambulatory surgery centers (2014), and outpatient hemodialysis facilities (2015), among other settings.^§§ ^LTC facilities currently are not covered by CMS quality reporting requirements. LTC employers can use the LTC web-based toolkit^¶¶^ developed by CDC and the National Vaccine Program Office, which provides access to resources, strategies, and educational materials for increasing influenza vaccination among HCP in long-term care settings.

**Summary**

**What is already known about this topic?**

The Advisory Committee on Immunization Practices recommends annual influenza vaccination for all health care personnel (HCP) to reduce influenza-related morbidity and mortality in health care settings. For the 2015–16 influenza season, the estimated overall influenza vaccination coverage among health care personnel was 79.0%.

**What is added by this report?**

Influenza vaccination coverage among HCP during the 2016–17 influenza season, assessed using an opt-in Internet panel survey, was 78.6%, similar to coverage during the 2015–16 season. Employer vaccination requirements and offering vaccination at the workplace at no cost were associated with higher vaccination coverage. Occupational settings with the lowest influenza vaccination coverage were the least likely to require vaccination or provide vaccination on-site at no cost.

**What are the implications for public health practice?**

Employer vaccination requirements or, in the absence of requirements, offering influenza vaccination on-site at no cost, can achieve high HCP vaccination coverage. Implementing comprehensive evidence-based worksite intervention strategies is important to ensure HCP and patients are protected against influenza.

## Footnotes

* Physicians, nurse practitioners, physician assistants, nurses, dentists, pharmacists, allied health professionals, technicians, and technologists were recruited from the current membership roster of Medscape. Additional information on Medscape is available at http://www.medscape.com.

† Assistants, aides, and nonclinical personnel (e.g., administrators, clerical support workers, janitors, food service workers, and housekeepers) were recruited from general population Internet panels operated by Survey Sampling International. Additional information on Survey Sampling International and its incentives for online survey participants is available at https://www.surveysampling.com.

§ Population control totals of U.S. health care personnel by occupation and work setting were obtained from the Bureau of Labor Statistics, U.S. Department of Labor, Occupational Employment Statistics, May 2015 National Industry-Specific Occupational Employment and Wage Estimates (https://www.bls.gov/oes/current/oessrci.htm). Population control totals by other demographic characteristics were obtained from the U.S. Census Bureau, Current Population Survey Monthly Labor Force Data, September 2016 (https://www.bls.gov/cps/data.htm).

¶ Additional information on obstacles to inference in nonprobability samples is available at http://www.aapor.org/Education-Resources/Reports/Non-Probability-Sampling.aspx.

** A survey response rate requires specification of the denominator at each stage of sampling. During recruitment of an online opt-in survey sample, such as the Internet panels described in this report, these numbers are not available; therefore, a response rate cannot be calculated. Instead, the survey cooperation rate is provided.

†† Employer promoted influenza vaccination among employees through public identification of vaccinated persons, financial incentives or rewards to individual persons or groups of employees, competition between units or care areas, free or subsidized cost of vaccination, personal reminders to be vaccinated, or publicizing of the number or percentage of employees receiving vaccination.

¶¶ https://www.cdc.gov/flu/toolkit/long-term-care/index.htm.
